# Whole-Genome Comparison of Representatives of All Variants of SARS-CoV-2, Including Subvariant BA.2 and the GKA Clade

**DOI:** 10.1155/2023/6476626

**Published:** 2023-03-09

**Authors:** Ida B. K. Suardana, Bayu K. Mahardika, Made Pharmawati, Putu H. Sudipa, Tri K. Sari, Nyoman B. Mahendra, Gusti N. Mahardika

**Affiliations:** ^1^Virology Laboratory, The Faculty of Veterinary Medicine, Udayana University, Denpasar, Bali, Indonesia; ^2^The Animal Biomedical and Molecular Biology Laboratory, Udayana University, Jl. Sesetan-Markisa 6A, Denpasar 80223, Bali, Indonesia; ^3^The Biology Study Program, The Faculty of Mathematic and Natural Science, Udayana University, Kampus Bukit Jimbaran, Badung, Bali, Indonesia; ^4^Veterinary Bacteriology and Mycology Laboratory, The Faculty of Veterinary Medicine, Udayana University, Denpasar, Bali, Indonesia; ^5^The Department of Obstetrics and Genecology, The Faculty of Medicine, Udayana University, Kuta Selatan, Bali, Indonesia

## Abstract

Since its discovery at the end of 2019, severe acute respiratory syndrome coronavirus 2 (SARS-CoV-2) has rapidly evolved into many variants, including the subvariant BA.2 and the GKA clade. Genomic clarification is needed for better management of the current pandemic as well as the possible reemergence of novel variants. The sequence of the reference genome Wuhan-Hu-1 and approximately 20 representatives of each variant were downloaded from GenBank and GISAID. Two representatives with no track of in-definitive nucleotides were selected. The sequences were aligned using muscle. The location of insertion/deletion (indel) in the genome was mapped following the open reading frame (ORF) of Wuhan-Hu-1. The phylogeny of the spike protein coding region was constructed using the maximum likelihood method. Amino acid substitutions in all ORFs were analyzed separately. There are two indel sites in ORF1AB, eight in spike, and one each in ORF3A, matrix (MA), nucleoprotein (NP), and the 3′-untranslated regions (3′UTR). Some indel sites and residues/substitutions are not unique, and some are variant-specific. The phylogeny shows that Omicron, Deltacron, and BA2 are clustered together and separated from other variants with 100% bootstrap support. In conclusion, whole-genome comparison of representatives of all variants revealed indel patterns that are specific to SARS-CoV-2 variants or subvariants. Polymorphic amino acid comparison across all coding regions also showed amino acid residues shared by specific groups of variants. Finally, the higher transmissibility of BA.2 might be due at least in part to the 48 nucleotide deletions in the 3′UTR, while the seem-to-be extinction of GKA clade is due to the lack of genetic advantages as a consequence of amino acid substitutions in various genes.

## 1. Introduction

The rapid evolution of severe acute respiratory syndrome 2 virus (SARS-CoV-2), the causative agent of the coronavirus 2019 (COVID-19) pandemic, requires immediate scientific clarification to better manage the current pandemic and to serve as a reference for the possible emergence of novel variants. Since it was discovered at the end of 2019 [1], the original virus has evolved into many variants, which could be attributed to specific clinical consequences [2]. Established variants of concern (VOCs) are Alpha, Beta, Gamma, Delta, and Omicron; Lambda and Mu are variants of interest (VOIs), and GH/490R is a variant under monitoring (VUM). Some other clusters of viruses that are closely related to Omicron, the so-called BA.2 and GKA clades, which carry molecular markers of Delta and Omicron variants, require special attention. Originally, detection of the GKA clade, popularly known as Deltacron, led to arguments that it may be the result of a sequencing error [3]. However, there is an increasing number of SARS-CoV-2 whole-genome sequences labelled as clade GKA with the notification “this submission requires investigation! It appears to contain markers of multiple lineages from both Delta and Omicron variants” in the database.

The capacity to spread globally as well as other biological properties of each variant must be encoded, at least in part, by its genome. Whole-genome comparison should also be able to confirm the establishment of the BA.2 subvariant and GKA clade. Based on previous publications [4, 5], the whole genome organization of SARS-CoV-2 after the ORF annotation of Wuhan-Hu-1 and adding 5′- and 3′UTR and intergenic sequence (IGS), is 5′UTR-ORF1AB- IGS-Spike-IGS-ORF3A-ORF3B-IGS-Protein E-IGS-membrane (MA)-IGS-ORF6-ORF7A-ORF7B-ORF8-IGS-Nucleoprotein NP-ORF-10-3′UTR. Intergenic sequences (IGSs) have been identified previously [6–8].

Large submissions of whole genome sequences pose a major computational challenge, and some portions of submitted sequences contain a long track of nondefinitive nucleotides. Here, we identify insertion/deletion (indel) and amino acid substitution patterns in the whole genome of representative variants, including the BA.2 subvariant and the GKA clade.

## 2. Materials and Methods

The sequence of the reference genome of SARS-CoV-2 strain Wuhan-Hu-1 (accession number NC_045512) was downloaded from GenBank. Ten to 20 complete sequences of each definitive variant as well as the subvariant BA.2 and GKA clade were selected randomly from GISAID and downloaded. Two representatives of each variant with no undetermined nucleotide of “N” or other IUPAC nucleotide codes were selected. The dataset identifier is EPI_SET ID: EPI_SET_230223kx; doi: 10.55876/gis8.230223kx. Sequences with a single N or in-definitive nucleotide were accepted. The sequences were aligned using muscle in MEGA-X software [9]. The locations of deletions/insertions in the genome of SARS-CoV-2 were mapped following the open reading frame of Wuhan-Hu-1, as available in the GenBank file. ORFs were analyzed separately to determine the effects of mutations and deletion/insertion.

Using the corresponding ORF of the coding region of Wuhan-Hu-1, the first 15 nucleotides of the 5′-terminus were searched, and the sequence prior to the marked sequence was deleted. The last 15 nucleotides of Wuhan-Hu-1 were used. The selected sequences were translated into amino acid sequences and aligned using MEGA-X software [9]. Using the same software, the data were exported in Mega format and analyzed further for polymorphic amino acids. We identified amino acids that were consistently substituted from Wuhan-Hu-1 across all variants and amino acids shared by the Omicron, BA2, and GKA lineages, Omicron and BA.2, Delta and GKA, Delta, Omicron, BA.2, and GKA, as well as Omicron and GKA. The final fasta file of the data set is available in Supplementary Material 1.

The phylogeny of the spike protein coding region of the representatives of variants was constructed using the maximum likelihood method and JTT matrix-based model [10] conducted in MEGA-X software [9]. The phylogenetic tree was rooted to Wuhan-Hu-1 sequence.

## 3. Results

The indel pattern and its location in the whole genome of representatives of various variants of SARS-CoV-2 are presented in Table 1. There are two indel sites in ORF1AB, eight in spike, and one each in ORF3A, MA, NP, and 3′UTR. No indel occurs in IGS. Some indel sites are not unique, as they occur in more than one variant. D21605-21613 is unique to Omicron and DA.2 variants, and D21965-21967 is unique to the Alpha variant; I21968-21971 and D26143-26146 are unique to the Mu variant, and D28351-28359 is unique to the Omicron and DA.2 variants; and D29723-29748 is unique to the DA.2 variant. Some indels occur in one representative of the variant.

All polymorphic amino acids of all proteins of two representatives of each variant of SARS-CoV-2 are listed in Supplementary Material 2. A summary of unique amino acids across entire genes in at least one of the representative strains of SARS-CoV-2 variants is presented in Table 2. Amino acids consistently substituted from Wuhan-Hu-1 across all variants are ORF1AB P4715L/F and spike D618G. Regarding the amino acids shared by Omicron, BA2, and GKA, there are 10 in ORF1AB, 21 in spike, and one each in ORF3A, ORF6, and NP. Three deletions in NP are unique to Omicron and BA.2. Exclusive to Delta and GKA are two deletions in ORF1AB, four in spike, three in NP, and two in ORF7A and ORF8; spike amino acids shared by Delta, Omicron, BA.2, and GKA occur only once. Three insertions, Ins216E, Ins217P, and Ins218E, are unique to Omicron and GKA. GH/490 harbors 16 variants specific to Wuhan-Hu-1, namely, five in ORF1AB, eight in spike, two in NP, and one in ORF3A. Unique amino acid substitutions from Wuhan-Hu-1 to GKA clade were nine amino acids in ORF1AB, namely, E352D, A1306S, P2046L, A2529V, I2820V, V2930L, T3646A, P4715F, and A6319V, one in NP, namely, G215C, two in ORF3A, namely, S92L and D155Y, and one in ORF7B of T40I.

**Figure 1:**
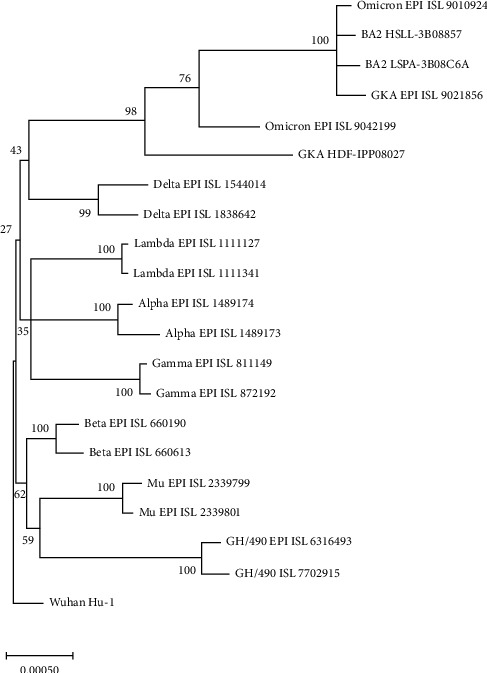
The phylogeny of the spike protein coding region of the representatives of variants of SARS-CoV-2. The phylogeny was constructed using the maximum likelihood method and JTT matrix-based model [10] conducted in MEGA-X software [9]. The phylogenetic tree was rooted to Wuhan-Hu-1 sequence. The tree with the highest log likelihood is shown. The percentage of trees in which the associated taxa clustered together is shown next to the branches.

The topology of phylogenetic analysis of the spike protein gene of two representatives of each variant of SARS-CoV-2 is presented in Figure 1. The phylogeny shows that Omicron, Deltacron, and BA2 are clustered together and separated from other variants with 100% bootstrap support.

## 4. Discussion

The genetic diversity of coronaviruses occurs through mutation and recombination, as it has been described for SARS-CoV-2 too [11]. Although the RNA-dependent RNA polymerases of coronaviruses possess proof-reading capacity [12], the virus still undergoes mutation, which might lead to amino acid replacement. Such changes impact the biology of the virus as well as the clinical manifestation of its infection. Recombination involves viral RNA merging with other RNAs, either its own RNA, the RNA of other viruses, or cellular RNA; thus, template switching occurs during transcription [13]. This process leads to RNA indels. Mutations in SARS-CoV-2 prior to the emergence of variants have been reported [6]. In HIV, the deletions occurred by at least three different mechanisms: (i) misalignment of the growing point; (ii) incorrect synthesis and termination in the primer-binding sequence during the synthesis of the plus-strand strong-stop DNA; and (iii) incorrect synthesis and termination before the primer-binding sequence during synthesis of the plus-strand strong-stop DNA [14].

Previous whole-genome comparisons have been conducted, including for Omicron [15]. However, that work focused on phylogeny and did not cover the recently identified BA.2 and GPA lineages, which are colloquially known as Deltacron. Indels and amino acid substitutions unique to specific variants were not described.

Through random selection of variant representatives with definitive sequences across the genome, we managed to identify unique patterns of indels and amino acid substitutions. Even with only two representatives for each variant, which is indeed the limitation of this study, we identified quasispecies or, in the case of a variant, quasivariant. Viral quasispecies refers to a population structure that consists of extremely large numbers of variant genomes, termed mutant spectra, mutant swarms, or mutant clouds [16]. For SARS-CoV-2, this phenomenon has been discovered even in single infected individuals [17–21]. We proposed the term quasivariant, as many indels and amino acid substitutions occur in one of only two representatives. We believe that we will find more variation if we analyze more variant representatives.

Amino acids consistently substituted from Wuhan-Hu-1 across all variants are ORF1AB P4715L/F and spike D618G. The D618G has been covered in previous works [6, 22–29]. ORF1AB P4715L/F has also been described [30, 31]. A database-wide survey is needed to understand the frequency of those substitutions.

The variant that harbors the most variant-specific substitution from Wuhan-Hu-1 is VUM GH/490. Both representatives show five, eight, two, and one amino acid substitutions in ORF1AB, spike, NP, and ORF3A, respectively. This VUM is being tracked in Europe, Africa, Asia, and America; however, the genome frequency for access of GISAID dated March 30, 2022, is lower than 0.3%.

The GKA clade does not comprise a unique variant. It harbors no unique indels or substitutions compared with Wuhan-Hu-1, but it does share 34 amino acid replacements from Wuhan-Hu-1 with Omicron and BA2, 13 with Delta, one in spike with Delta, Omicron, and BA.2. Three insertions in the spike in one representative of Ins216E, Ins217P, and Ins218E of the GKA clade are shared with Omicron. The molecular signatures of Delta and Omicron are obvious in the GKA clade. It is plausible that the GKA clade is an Omicron subvariant. We suggest that the clade is not the result of sequencing errors, as previously thought [3].

The GKA clade seems to have no genetic advantage, so it becomes extinct shortly after its discovery. There are only 89 full genome sequences tagged with GKA clade upon access to the GISAIS database on May 3^rd^, 2022. The collection date of the earliest sequence was dated on January 20, 2022, and the last one was dated on March 21, 2022. This clade poses nine amino acid changes from the reference strain of Wuhan-Hu-1 in ORF1AB, two in ORF3A, and one in NP and ORF7B. No unique amino acid change was observed in the spike protein. The clade seems to be suppressed by antibodies to other variants following previous natural infection and/or vaccination.

Interestingly, we identified a truncated ORF3A in the Mu variant. Deletion of four nucleotides generates a stop codon; thus, ORF3A in this variant is 257 amino acids in length, whereas the others are 275 residues long. This accessory protein contributes to the pathogenesis of SARS-CoV-2 by inducing pathological apoptosis [32]. The effect of the Mu variant at the cellular level has not yet been described. One article on this variant covered the neutralization effect of antibodies [33]. According to the GISAID database accessed on March 30, 2022, this variant has been identified in many countries, with a maximum global genome frequency of less than 1%, which has declined recently.

BA.2 differs from Omicron in the deletion of 48 nucleotides from the 3′UTR. The 3′UTR of coronaviruses contains all cis-acting sequences necessary for viral replication and binds to cellular as well as the viral components nsp1 and N proteins [34], which are required for minus-strand RNA synthesis [35]. This has also been described in SARS-CoV-2, whereby the 3′UTR is involved in genomic dimerization and interacts with cellular microRNA [36]. BA.2 has recently increased in frequency in multiple regions of the world, suggesting that it has a selective advantage over Omicron [37–40]. The genome frequency of BA.2 has increased exponentially to 90% of total Omicron submissions, as based on GISAID accessed on the previous date. As the original SARS-CoV-2 has a basic reproduction number (R0) of 2.4-3 [41], Delta has an R0 of 5 [42], and Omicron has an R0 of estimated to be higher than 10 or three times greater than Delta [43]; additionally, BA.2 subvariant might have an R0 of 15 or higher. The higher transmissibility of BA.2 might be attributed, at least in part, to the shorter 3′UTR, which results in a higher speed of viral replication, which needs to be investigated further. However, because the coding region across the whole genome, particularly for the spike protein of BA.2, is very close to that of Omicron, people who survived Omicron infection should be naturally protected against BA.2.

Phylogenetic analysis (Figure 1) demonstrated that the BA2 and GKA subvariants are Omicron variant. The phylogeny shows that Omicron, GKA/Deltacron, and BA2 are clustered together and separated from other variants with 100% bootstrap support.

## 5. Conclusion

Whole-genome comparison of representatives of all variants revealed indel patterns that are specific to SARS-CoV-2 variants or subvariants. Polymorphic amino acid comparison across all coding regions also showed amino acid residues shared by specific groups of variants. Finally, the higher transmissibility of BA.2 might be due at least in part to the 48 nucleotide deletions in the 3′UTR, which result in a higher speed of viral replication, while the seem-to-be extinction of GKA clade is due to the lack of genetic advantage as a consequence of amino acid substitutions in various genes.

## Figures and Tables

**Table 1:** Insertion/deletion pattern and its location at the whole genome of representatives of various variants of SARS-CoV-2.

No	Position^*∗*^	D6485-6487	D11260-11268	D21605-21613	D21959-21967	D21965-21967	I21968-21971	D22005-22010	D22170-22172	I22181-22189	D22266-22274	D26143-26146	D27055-27067	D28351-28359	D29723-29748
	Location at the genome^*∗∗*^	ORF1AB	ORF1AB	Spike	Spike	Spike	Spike	Spike	Spike	Spike	Spike	ORF3A	MA	NP	3′UTR
	Virus strain
1	WH-1	−	−	−	−	−	−	−	−	−	−	−	−	−	−
2	A-1	−	+	−	−	+	−	−	−	−	−	−	−	−	−
3	A-2	−	+	−	−	+	−	−	−	−	−	−	−	−	−
4	B-1	−	+	−	−	−	−	−	−	−	+	−	−	−	−
5	B-2	−	+	−	−	−	−	−	−	−	−	−	−	−	−
6	G-1	−	+	−	−	−	−	−	−	−	−	−	−	−	−
7	G2	−	+	−	−	−	−	−	−	−	−	−	−	−	−
8	D-1	−	−	−	−	−	−	+	−	−	−	−	−	−	−
9	D2	−	−	−	−	−	−	−	−	−	−	−	+	−	−
10	L-1	−	−	−	−	−	−	−	−	−	−	−	−	−	−
11	L-2	−	−	−	−	−	−	−	−	−	−	−	−	−	−
12	M-1	−	−	−	−	−	+	−	−	−	−	+	−	−	−
13	M-2	−	−	−	−	−	+	−	−	−	−	+	−	−	−
14	O-1	−	+	+	−	−	−	−	−	−	−	−	−	+	−
15	O-2	+	+	−	+	−	−	−	+	+	−	−	−	+	−
16	BA2-1	−	+	+	−	−	−	−	+	+	−	−	−	+	+
17	BA2-2	−	+	+	−	−	−	−	−	−	−	−	−	+	+
18	GKA-1	−	−	−	−	−	−	−	−	−	−	−	−	−	−
19	GKA-2	−	−	−	−	−	−	+	−	−	−	−	−	−	−
20	GH-1	−	−	−	−	−	−	−	−	−	−	−	−	−	−
21	GH-2	−	−	−	−	−	−	−	−	−	−	−	−	−	−

N: no insertion/deletion; P: positive insertion/deletion; ^*∗*^Position based on aligned sequence starting from nucleotide no. 1 of Wuhan-Hu-1; ^*∗∗*^Location in the genome was based on GenBank data of Wuhan-Hu-1; Virus strains and GISAID codes WH-1 is Wuhan-Hu-1, A-1 is Alpha EPI ISL 1489174, A-2 is Alpha EPI ISL 1489173, B-1 is Beta EPI ISL 660190, B-2 is Beta EPI ISL 660613, G-1 is Gamma EPI ISL 811149, G-2 is Gamma EPI ISL 87219, D-1 is Delta EPI ISL 1544014, D-2 is Delta EPI ISL 1838642, L-1 is Lambda EPI ISL 1111127, L-2 is Lambda EPI ISL 1111341, M-1 is Mu EPI ISL 2339799, M-2 is Mu EPI ISL 2339801, O-1 is Omicron EPI ISL 9010924, O-2 is Omicron EPI ISL 9042199, BA2-1 is BA2 LSPA-3B08C6A, BA2-2 is BA2 HSLL-3B08857, GKA-1 is EPI ISL 9021856, GKA-2 is HDF-IPP08027, GH-1 is GH/490 EPI ISL 6316493, and GH-2 is GH/490 ISL 7702915.

**Table 2:** Unique amino acids across whole genes in at least one of the representing strains of various variants of SARS-CoV-2.

Consistent substitution to Wuhan-Hu-1	Specific for omicron, BA2, and GKA	Specific for omicron and BA2	Specific for delta and GKA	Specific to delta, omicron, BA.2, and GKA	Specific to omicron and GKA	Unique to GH	Unique to GKA clade
ORF1AB (1)	ORF1AB (10)		ORF1AB (2)			ORF1AB (5)	ORF1AB (9)
P4715L/F	S135R, T842I, G1307S, L3027F, T3090I, L3201F, P3395H, R5716C, I5967V, T6594I		G5063S, P5401L			E452G, L2119F, A2355S, S3149F, R3164H	E352D, A1306S, P2046L, A2529V, I2820V, V2930L T3646A, P4715F, A6319V

Spike^*∗*^ (1)	Spike^*∗*^ (21)		Spike^*∗*^ (4)	Spike^*∗*^ (1)	Spike^*∗*^ (3)	Spike^*∗*^ (8)	NP (1)
D618G	T19I, A27S, G142D, V214G, G343D, S375F/L, S377P, S379F, T380A, D409N, R412S, N444K, E488A, Q497R, Q502R, Y509H, N683K, N768K, D800Y, Q958H, N973K		T19R, E157G, F158del, R159del	T482K	Ins216E, Ins217P, Ins218E	P9L, E96Q, I211T, R350S, N398S, Y453N, F494R, D940H	G215C

	NP (1)	NP (3)	NP (3)			NP (2)	ORF3A (2)
	S413R	E31del, R32del, S33del	D63G, R203M, D377Y			D22Y, E378Q	S92L, D155Y

	ORF3A (1)		ORF7A (2)			ORF3A (1)	ORF7B (1)
	T223I		V82A, T120I			T32I	T40I

	ORF6 (1)		ORF8 (2)				
	D61L		D119del, F120del				

Numbers in parentheses were the number of unique amino acids in the corresponding gene; numbering was based on ORFs of Wuhan-Hu-1, except for spike; ^*∗*^numbering in spike protein from 145 to 215 is Wuhan-Hu-1 numbering plus 1 due to insertion in Mu variant, while numbering of spike from 219 or higher is Wuhan-hu-1 numbering plus 4 due to the insertion of EPE residues in omicron.

## Data Availability

All genome sequences and associated metadata in this dataset are published in the GISAID's EpiCov database. The final dataset is available at GISAID with identifier EPI_SET_230223kx, doi: 10.55876/gis8.230223kx. To view the contributors and each individual sequence with details such as the accession number, virus name, collection date, originating lab and submitting lab, and the list of authors, we need to visit 10.55876/gis8.230223kx. All polymorphic amino acids of all proteins of two representatives of each variant of SARS-CoV-2 are listed in Supplementary Material 1.

## Supplementary Materials

Supplementary Materials All polymorphic amino acids of all proteins of two representatives of each variant of SARS-CoV-2.

## References

[1] Wang H., Li X., Li T. (2020). The genetic sequence, origin, and diagnosis of SARS-CoV-2. *European Journal of Clinical Microbiology and Infectious Diseases*.

[2] AlMalki F. A., Albukhaty S., Alyamani A. A., Khalaf M. N., Thomas S. (2022). The relevant information about the severe acute respiratory syndrome coronavirus 2 (SARS-CoV-2) using the five-question approach (when, where, what, why, and how) and its impact on the environment. *Environmental Science and Pollution Research International*.

[3] Kreier F. (2022). Deltacron: the story of the variant that wasn’t. *Nature*.

[4] Wu F., Zhao S., Yu B. (2020). A new coronavirus associated with human respiratory disease in China. *Nature*.

[5] Jin Y., Yang H., Ji W. (2020). Virology, epidemiology, pathogenesis, and control of COVID-19. *Viruses*.

[6] Mercatelli D., Giorgi F. M. (2020). Geographic and genomic distribution of SARS-CoV-2 mutations. *Frontiers in Microbiology*.

[7] Rahimi A., Mirzazadeh A., Tavakolpour S. (2021). Genetics and genomics of SARS-CoV-2: a review of the literature with the special focus on genetic diversity and SARS-CoV-2 genome detection. *Genomics*.

[8] Jungreis I., Sealfon R., Kellis M. (2021). SARS-CoV-2 gene content and COVID-19 mutation impact by comparing 44 Sarbecovirus genomes. *Nature Communications*.

[9] Kumar S., Stecher G., Li M., Knyaz C., Tamura K., Mega X. (2018). Mega X: molecular evolutionary genetics analysis across computing platforms. *Molecular Biology and Evolution*.

[10] Jones D. T., Taylor W. R., Thornton J. M. (1992). The rapid generation of mutation data matrices from protein sequences. *Bioinformatics*.

[11] Pollett S., Conte M. A., Sanborn M. (2021). A comparative recombination analysis of human coronaviruses and implications for the SARS-CoV-2 pandemic. *Scientific Reports*.

[12] Robson F., Khan K. S., Le T. K. (2020). Coronavirus RNA proofreading: molecular basis and therapeutic targeting. *Molecular Cell*.

[13] Kim M. J., Kao C. (2001). Factors regulating template switch in vitro by viral RNA-dependent RNA polymerases: implications for RNA-RNA recombination.

[14] Pulsinelli G. A., Temin H. M. (1991). Characterization of large deletions occurring during a single round of retrovirus vector replication: novel deletion mechanism involving errors in strand transfer. *Journal of Virology*.

[15] Kandeel M., Mohamed M. E. M., Abd El-Lateef H. M., Venugopala K. N., El-Beltagi H. S. (2021). Omicron variant genome evolution and phylogenetics. *Journal of Medical Virology*.

[16] Domingo E., Perales C. (2019). Viral quasispecies. *PLoS Genetics*.

[17] Sun F., Wang X., Tan S. (2021). SARS-CoV-2 quasispecies provides an advantage mutation pool for the epidemic variants. *Microbiology Spectrum*.

[18] Ghorbani A., Samarfard S., Ramezani A. (2020). Quasi-species nature and differential gene expression of severe acute respiratory syndrome coronavirus 2 and phylogenetic analysis of a novel Iranian strain. *Infection, Genetics and Evolution*.

[19] Lau B. T., Pavlichin D., Hooker A. C. (2020). Profiling SARS-CoV-2 mutation fingerprints that range from the viral pangenome to individual infection quasispecies. *medRxiv*.

[20] Armero A., Berthet N., Avarre J. C. (2021). Intra-host diversity of SARS-cov-2 should not Be neglected: case of the state of victoria, Australia. *Viruses*.

[21] Jary A., Leducq V., Malet I. (2020). Evolution of viral quasispecies during SARS-CoV-2 infection. *Clinical Microbiology and Infections*.

[22] Andres C., Pinana M., Borras-Bermejo B. (2022). A year living with SARS-CoV-2: an epidemiological overview of viral lineage circulation by whole-genome sequencing in Barcelona city (Catalonia, Spain). *Emerging Microbes and Infections*.

[23] Leung K., Pei Y., Leung G. M., Lam T. T., Wu J. T. (2021). Estimating the transmission advantage of the D614G mutant strain of SARS-CoV-2, December 2019 to June 2020. *Euro Surveillance*.

[24] Cheng Y. W., Chao T. L., Li C. L. (2021). D614G substitution of SARS-CoV-2 spike protein increases syncytium formation and virus titer via enhanced furin-mediated spike cleavage. *mBio*.

[25] Yang T. J., Yu P. Y., Chang Y. C., Hsu S. T. D. (2021). D614G mutation in the SARS-CoV-2 spike protein enhances viral fitness by desensitizing it to temperature-dependent denaturation. *Journal of Biological Chemistry*.

[26] Bhattacharya M., Chatterjee S., Sharma A. R., Agoramoorthy G., Chakraborty C. (2021). D614G mutation and SARS-CoV-2: impact on S-protein structure, function, infectivity, and immunity. *Applied Microbiology and Biotechnology*.

[27] Mat Yassim A. S., Asras M. F. F., Gazali A. M., Marcial-Coba M. S., Zainulabid U. A., Ahmad H. F. (2022). COVID-19 outbreak in Malaysia: decoding D614G mutation of SARS-CoV-2 virus isolated from an asymptomatic case in pahang. *Materials Today Proceedings*.

[28] Eaaswarkhanth M., Al Madhoun A., Al-Mulla F. (2020). Could the D614G substitution in the SARS-CoV-2 spike (S) protein be associated with higher COVID-19 mortality?. *International Journal of Infectious Diseases*.

[29] Muttineni R., Kammili N., Bingi T. C. (2021). Clinical and whole genome characterization of SARS-CoV-2 in India. *PLoS One*.

[30] De Marco C., Marascio N., Veneziano C. (2022). Whole-genome analysis of SARS-CoV-2 in a 2020 infection cluster in a nursing home of Southern Italy. *Infection, Genetics and Evolution*.

[31] Banerjee S., Seal S., Dey R., Mondal K. K., Bhattacharjee P. (2021). Mutational spectra of SARS-CoV-2 orf1ab polyprotein and signature mutations in the United States of America. *Journal of Medical Virology*.

[32] Ren Y., Shu T., Wu D. (2020). The ORF3a protein of SARS-CoV-2 induces apoptosis in cells. *Cellular and Molecular Immunology*.

[33] Uriu K., Kimura I., Shirakawa K. (2021). Genotype to phenotype Japan C: neutralization of the SARS-CoV-2 Mu variant by convalescent and vaccine serum. *New England Journal of Medicine*.

[34] Yang D., Leibowitz J. L. (2015). The structure and functions of coronavirus genomic 3’ and 5’ ends. *Virus Research*.

[35] Lin Y. J., Zhang X., Wu R. C., Lai M. M. (1996). The 3’ untranslated region of coronavirus RNA is required for subgenomic mRNA transcription from a defective interfering RNA. *Journal of Virology*.

[36] Imperatore J. A., Cunningham C. L., Frye C. J., Pellegrene K. A., Evanseck J. D., Mihailescu M.-R. (2021). Conserved elements in the 3’-UTR of SARS-CoV-2: involvement in genomic dimerization and interactions with cellular micrornas. *Biophysical Journal*.

[37] Yu J., Collier A. r Y., Rowe M. (2022). Comparable neutralization of the SARS-CoV-2 Omicron BA.1 and BA.2 variants. *medRxiv*.

[38] Cheng V. C. C., Ip J. D., Chu A. W. H. (2022). Rapid spread of severe acute respiratory syndrome coronavirus 2 (SARS-CoV-2) Omicron subvariant BA.2 in a single-source community outbreak. *Clinical Infectious Diseases*.

[39] Mahase E. (2022). Omicron sub-lineage BA.2 may have “substantial growth advantage”, UKHSA reports. *BMJ*.

[40] Desingu P. A., Nagarajan K., Omicron B. A. (2022). Omicron BA.2 lineage spreads in clusters and is concentrated in Denmark. *Journal of Medical Virology*.

[41] D’Arienzo M., Coniglio A. (2020). Assessment of the SARS-CoV-2 basic reproduction number, R 0, based on the early phase of COVID-19 outbreak in Italy. *Biosafety and Health*.

[42] Liu Y., Rocklov J. (2021). The reproductive number of the Delta variant of SARS-CoV-2 is far higher compared to the ancestral SARS-CoV-2 virus. *Journal of Travel Medicine*.

[43] Ito K., Piantham C., Nishiura H. (2022). Relative instantaneous reproduction number of Omicron SARS-CoV-2 variant with respect to the Delta variant in Denmark. *Journal of Medical Virology*.

