# Supplementary material for: Whole-Genome Comparison of Representatives of All Variants of SARS-CoV-2, Including Subvariant BA.2 and the GKA Clade
Source: Adv Virol. 2023 Mar 9;2023:6476626. doi: 10.1155/2023/6476626 (PMC10019969; doi:10.1155/2023/6476626)
Supplement: Supplementary Materials — All polymorphic amino acids of all proteins of two representatives of each variant of SARS-CoV-2. [file 6476626.f1.pdf]

All polymorphic amino acids of all proteins of two representatives of each variant of SARS-CoV-2

Protein	Pos	WH-1	A-1	A-2	B-1	B-2	G-1	G2	D-1	D2	L-1	L-2	M-1	M-2	O-1	O-2	BA2-1	BA2-2	GKA-1	GKA-2	GH-1	GH-2
ORF1AB	12	T	.	.	.	.	.	.	.	.	.	.	.	.	.	.	.	.	.	.	.	I
	62	P	.	.	.	.	.	.	.	.	.	.	.	A	.	.	.	.	.	.	.	.
	117	A	.	.	.	.	.	.	.	.	.	.	.	.	.	.	.	T	.	.	.	.
	135	S	.	.	.	.	.	.	.	.	.	.	.	.	R	.	R	R	R	.	.	.
	265	T	.	.	I	I	.	.	.	.	.	.	.	.	.	.	.	.	.	.	.	.
	306	V	.	.	.	.	.	.	.	.	.	.	I	.	.	.	.	.	.	.	.	.
	309	P	.	.	.	.	.	.	L	L	.	.	.	.	.	.	.	.	.	.	L	L
	352	E	.	.	.	.	.	.	.	.	.	.	.	.	.	.	.	.	.	D	.	.
	374	H	.	.	.	.	.	.	.	Y	.	.	.	.	.	.	.	.	.	.	.	.
	452	E	.	.	.	.	.	.	.	.	.	.	.	.	.	.	.	.	.	.	G	G
	842	T	.	.	.	.	.	.	.	.	.	.	.	.	I	.	I	I	I	.	.	.
	856	K	.	.	.	.	.	.	.	.	.	.	.	.	.	R	.	.	.	.	.	.
	1001	T	I	I	.	.	.	.	.	.	.	.	.	.	.	.	.	.	.	.	.	.
	1056	T	.	.	.	.	.	.	.	.	.	.	A	A	.	.	.	.	.	.	.	.
	1180	K	.	.	.	.	.	.	.	.	.	.	.	.	.	.	.	T	.	.	.	.
	1188	S	.	.	.	.	L	L	.	.	.	.	.	.	.	.	.	.	.	.	.	.
	1213	P	.	.	.	.	.	.	.	.	.	.	.	.	.	.	L	.	.	.	.	.
	1246	T	.	.	.	.	.	.	.	.	I	I	.	.	.	.	.	.	.	.	.	.
	1306	A	.	.	.	.	.	.	.	.	.	.	.	.	.	.	.	.	.	S	.	.
	1307	G	.	.	.	.	.	.	.	.	.	.	.	.	S	.	S	S	S	.	.	.
	1468	S	.	.	.	A	.	.	.	.	.	.	.	.	.	.	.	.	.	.	.	.
	1538	T	.	.	.	.	.	.	.	.	.	.	I	I	.	.	.	.	.	.	.	.
	1640	P	.	.	.	.	.	.	L	L	.	.	.	.	.	.	.	.	.	.	.	.
	1655	K	.	.	N	N	.	.	.	.	.	.	.	.	.	.	.	.	.	.	.	.
	1708	A	D	D	.	.	.	.	.	.	.	.	.	.	.	.	.	.	.	.	.	.
	1794	T	.	.	.	.	.	.	.	.	.	.	.	.	.	.	I	.	.	.	.	.
	1795	K	.	.	.	.	Q	Q	.	.	.	.	.	.	.	.	.	.	.	.	.	.
	1853	L	.	F	.	.	.	.	.	.	.	.	.	.	.	.	.	.	.	.	.	.
	1854	T	.	.	.	.	.	.	.	.	.	.	.	.	.	.	.	.	.	.	.	I
	2046	P	.	.	.	.	.	.	.	.	.	.	.	.	.	.	.	.	.	L	.	.
	2083	S	.	.	.	.	.	.	.	.	.	.	.	.	.	-	.	.	.	.	.	.
	2119	L	.	.	.	.	.	.	.	.	.	.	.	.	.	.	.	.	.	.	F	F
	2200	K	.	.	.	Q	.	.	.	.	.	.	.	.	.	.	.	.	.	.	.	.
	2201	N	.	.	.	Y	.	.	.	.	.	.	.	.	.	.	.	.	.	.	.	.

2230	I	T	T	.	.	.	.	.	.	.	.	.	.	.	.	.	.	.	.	.	.
2235	L	.	.	.	.	.	.	.	.	.	.	.	.	.	.	.	.	.	.	.	.
2287	P	.	.	.	.	.	.	.	.	S	S	.	.	.	.	.	.	.	S	.	.
2355	A	.	.	.	.	.	.	.	.	.	.	.	.	.	.	.	.	.	.	S	S
2376	P	.	.	.	.	.	.	.	.	.	.	.	.	.	.	.	.	L	.	.	.
2387	F	.	.	.	.	.	.	.	.	V	V	.	.	.	.	.	.	.	.	.	.
2500	S	F	.	.	.	.	.	.	.	.	.	.	.	.	.	.	.	.	.	.	.
2529	A	.	.	.	.	.	.	.	.	.	.	.	.	.	.	.	.	.	V	.	.
2611	T	.	.	.	.	.	.	.	.	.	.	.	.	.	I	.	.	.	.	.	.
2710	A	.	.	.	.	.	.	.	.	.	.	.	.	.	T	.	.	.	.	.	.
2797	S	.	.	.	F	.	.	.	.	.	.	.	.	.	.	.	.	.	.	.	.
2820	I	.	.	.	.	.	.	.	.	.	.	.	.	.	.	.	.	.	V	.	.
2930	V	.	.	.	.	.	.	.	.	.	.	.	.	.	.	.	.	.	L	.	.
2980	D	.	.	.	.	.	.	.	N	.	.	.	.	.	.	.	.	.	.	.	.
3027	L	.	.	.	.	.	.	.	.	.	.	.	.	F	.	F	F	F	.	.	.
3090	T	.	.	.	.	.	.	.	.	.	.	.	.	I	.	I	I	I	.	.	.
3116	L	.	.	.	.	.	F	.	.	.	.	.	.	.	.	.	.	.	.	.	.
3138	F	.	.	.	.	.	.	.	S	.	.	.	.	.	.	.	.	.	.	.	.
3149	S	.	.	.	.	.	.	.	.	.	.	.	.	.	.	.	.	.	.	F	F
3164	R	.	.	.	.	.	.	.	.	.	.	.	.	.	.	.	.	.	.	H	H
3201	L	.	.	.	.	.	.	.	.	P	P	.	.	F	.	F	F	F	.	.	.
3209	A	.	.	.	.	.	.	V	.	.	.	.	.	.	.	.	.	.	.	.	.
3255	T	.	.	.	.	.	.	.	.	I	I	I	I	I	I	I	I	I	I	I	I
3278	G	.	.	.	.	.	.	.	.	S	S	.	.	.	.	.	.	.	.	.	.
3353	K	.	.	R	R	.	.	.	.	.	.	.	.	.	.	.	.	.	.	.	.
3395	P	.	.	.	.	.	.	.	.	.	.	.	.	H	H	H	H	H	.	.	.
3580	H	.	.	.	.	.	.	.	Q	.	.	.	.	.	.	.	.	.	.	.	.
3646	T	.	.	.	.	.	.	.	.	.	.	.	.	.	.	.	.	.	A	.	.
3674	L	.	.	.	.	.	.	.	.	.	.	.	.	.	F	.	.	.	.	.	.
3675	S	-	-	-	-	-	-	.	.	.	.	.	.	-	-	-	-	.	.	.	.
3676	G	-	-	-	-	-	-	.	.	.	.	.	.	-	-	-	-	.	.	.	.
3677	F	-	-	-	-	-	-	.	.	L	?	.	.	-	-	-	-	L	.	.	.
3718	V	.	.	.	.	.	.	A	.	.	.	.	.	.	.	.	.	.	.	A	A
3729	Q	.	.	.	.	.	.	.	.	.	.	R	R	.	.	.	.	.	.	.	.
3758	I	.	.	.	.	.	.	.	.	.	.	.	.	.	V	.	.	.	.	.	.
3833	N	.	.	.	.	.	.	.	.	.	.	.	.	.	.	.	.	.	.	.	.
4483	K	.	R	.	.	.	.	.	.	.	.	.	.	.	.	.	.	.	.	.	.

	4715	P	L	L	L	L	L	L	L	L	L	L	L	L	L	L	L	L	L	F	L	L
	5063	G	.	.	.	.	.	.	S	.	.	.	.	.	.	.	.	.	.	S	.	.
	5360	S	.	.	.	.	.	.	.	.	.	.	.	.	.	.	.	P	.	.	.	.
	5401	P	.	.	.	.	.	.	L	.	.	.	.	.	.	.	.	.	.	L	.	.
	5496	P	.	.	.	.	.	.	.	.	.	.	H	.	.	.	.	.	.	.	.	.
	5540	T	.	.	.	.	N	.	.	.	.	.	.	.	.	.	.	.	.	.	.	.
	5665	E	.	.	.	.	D	D	.	.	.	.	.	.	.	.	.	.	.	.	.	.
	5716	R	.	.	.	.	.	.	.	.	.	.	.	.	C	.	C	C	C	.	.	.
	5743	P	.	.	.	.	.	.	.	.	.	.	S	S	.	.	.	.	.	.	.	.
	5967	I	.	.	.	.	.	.	.	.	.	.	.	.	V	V	V	V	V	.	.	.
	6175	T	.	.	.	.	.	.	.	.	.	.	.	.	.	.	I	.	.	.	.	.
	6319	A	.	.	.	.	.	.	.	.	.	.	.	.	.	.	.	.	.	V	.	.
	6564	T	.	.	.	.	.	.	.	.	.	.	.	.	I	.	I	I	I	.	.	.
	6686	H	.	.	.	.	.	.	Y	.	.	.	.	.	.	.	.	.	.	.	.	.
	6711	K	.	.	.	.	.	.	.	R	.	.	.	.	.	.	.	.	.	.	.	.
Spike	9	P	.	.	.	.	.	.	.	.	.	.	.	.	.	.	.	.	.	.	L	L
	18	L	.	.	.	.	F	F	.	.	.	.	.	.	.	.	.	.	.	.	.	.
	19	T	.	.	.	.	.	.	R	R	.	.	.	.	I	.	I	I	I	R	.	.
	20	T	.	.	.	.	N	N	.	.	.	.	.	.	.	.	.	.	.	.	.	.
	24	L	.	.	.	.	.	.	.	.	.	.	.	.	-	.	-	-	.	.	.	.
	25	P	.	.	.	.	.	.	.	.	.	.	.	.	-	.	-	-	.	.	.	.
	26	P	.	.	.	.	S	S	.	.	.	.	.	.	-	.	-	-	.	.	.	.
	27	A	.	.	.	.	.	.	.	.	.	.	.	.	S	.	S	S	.	S	.	.
	67	A	.	.	.	.	.	.	.	.	.	.	.	.	.	V	.	.	.	.	.	.
	69	H	-	-	.	.	.	.	.	.	.	.	.	.	.	-	.	.	.	.	.	.
	70	V	-	-	.	.	.	.	.	.	.	.	.	.	.	-	.	.	.	.	.	.
	75	G	.	.	.	.	.	.	.	.	V	V	.	.	.	.	.	.	.	.	.	.
	76	T	.	.	.	.	.	.	.	.	I	I	.	.	.	.	.	.	.	.	.	.
	80	D	.	.	A	A	.	.	.	.	.	.	.	.	.	.	.	.	.	.	.	.
	95	T	.	.	.	.	.	.	.	.	.	.	I	I	.	I	.	.	.	I	.	.
	96	E	.	.	.	.	.	.	.	.	.	.	.	.	.	.	.	.	.	.	Q	Q
	138	D	.	.	.	.	Y	Y	.	.	.	.	.	.	.	.	.	.	.	.	.	.
	142	G	.	.	.	.	.	.	.	.	.	.	.	.	D	D	D	D	D	D	.	D
	143	V	.	.	.	.	.	.	.	.	.	.	.	.	.	-	.	.	.	.	.	.
	144	-	-	-	-	-	-	-	-	-	-	-	T	T	-	-	-	-	-	-	-	-
	145	Y	-	-	.	.	.	.	.	.	.	.	L	L	.	-	.	.	.	.	F	.
	146	Y	.	.	.	.	.	.	.	.	.	.	N	N	.	-	.	.	.	.	.	.

157	E	.	.	.	.	.	.	G	.	.	.	.	.	.	.	.	.	.	G	.	.
158	F	.	.	.	.	.	.	-	.	.	.	.	.	.	.	.	.	.	-	.	.
159	R	.	.	.	.	.	.	-	.	.	.	.	.	.	.	.	.	.	-	.	.
191	R	.	.	.	.	S	S	.	.	.	.	.	.	.	.	.	.	.	.	S	S
211	I	.	.	.	.	.	.	.	.	.	.	.	.	.	.	.	.	.	.	T	T
212	N	.	.	.	.	.	.	.	.	.	.	.	.	-	.	.	.	.	-	.	.
213	L	.	.	.	.	.	.	.	.	.	.	.	.	.	I	.	.	.	I	.	.
214	V	.	.	.	.	.	.	.	.	.	.	.	.	G	.	G	G	G	.	.	.
216	-	-	-	-	-	-	-	-	-	-	-	-	-	-	E	-	-	-	E	-	-
217	-	-	-	-	-	-	-	-	-	-	-	-	-	-	P	-	-	-	P	-	-
218	-	-	-	-	-	-	-	-	-	-	-	-	-	-	E	-	-	-	E	-	-
219	D	.	.	G	.	.	.	.	.	.	.	.	.	.	.	.	.	.	.	.	.
343	G	.	.	.	.	.	.	.	.	.	.	.	.	D	D	D	D	D	D	.	.
245	L	.	.	-	.	.	.	.	.	.	.	.	.	.	.	.	.	.	.	.	.
246	L	.	.	-	.	.	.	.	.	.	.	.	.	.	.	.	.	.	.	.	.
247	A	.	.	-	.	.	.	.	.	.	.	.	.	.	.	.	.	.	.	.	.
350	R	.	.	.	.	.	.	.	.	.	.	K	K	.	.	.	.	.	.	S	S
375	S	.	.	.	.	.	.	.	.	.	.	.	.	F	L	F	F	F	L	.	.
377	S	.	.	.	.	.	.	.	.	.	.	.	.	P	P	P	P	P	P	.	.
379	S	.	.	.	.	.	.	.	.	.	.	.	.	F	F	F	F	F	F	.	.
380	T	.	.	.	.	.	.	.	.	.	.	.	.	A	.	A	A	A	.	.	.
398	N	.	.	.	.	.	.	.	.	.	.	.	.	.	.	.	.	.	.	S	S
409	D	.	.	.	.	.	.	.	.	.	.	.	.	N	.	N	N	N	.	.	.
412	R	.	.	.	.	.	.	.	.	.	.	.	.	S	.	S	S	S	.	.	.
421	K	.	.	N	.	T	T	.	.	.	.	.	.	N	N	N	N	N	N	.	.
444	N	.	.	.	.	.	.	.	.	.	.	.	.	?	K	K	K	K	K	.	.
450	G	.	.	.	.	.	.	.	.	.	.	.	.	.	S	.	.	.	S	.	.
453	Y	.	.	.	.	.	.	.	.	.	.	.	.	.	.	.	.	.	.	N	N
456	L	.	.	.	.	.	.	R	R	Q	Q	.	.	.	.	.	.	.	.	.	.
481	S	.	N	.	.	.	.	.	.	.	.	.	.	N	N	N	N	N	N	.	.
482	T	.	.	.	.	.	.	K	K	.	.	.	.	K	K	K	K	K	K	.	.
488	E	.	.	K	K	K	K	.	.	.	.	K	K	A	A	A	A	A	A	.	.
494	F	.	.	.	.	.	.	.	.	S	S	.	.	.	.	.	.	.	.	R	R
497	Q	.	.	.	.	.	.	.	.	.	.	.	.	R	R	R	R	R	R	.	.
500	G	.	.	.	.	.	.	.	.	.	.	.	.	.	S	.	.	.	S	.	.
502	Q	.	.	.	.	.	.	.	.	.	.	.	.	R	R	R	R	R	R	.	.
505	N	Y	Y	Y	Y	Y	Y	.	.	.	.	Y	Y	Y	Y	Y	Y	Y	Y	Y	Y

	509	Y	.	.	.	.	.	.	.	.	.	.	.	.	H	H	H	H	H	H	.	.
	551	T	.	.	.	.	.	.	.	.	.	.	.	.	.	K	.	.	.	K	.	.
	574	A	D	D	.	.	.	.	.	.	.	.	.	.	.	.	.	.	.	.	.	.
	618	D	G	G	G	G	G	G	G	G	G	G	G	G	G	G	G	G	G	G	G	G
	626	V	A	.	.	.	.	.	.	.	.	.	.	.	.	.	.	.	.	.	.	.
	659	H	.	Y	.	.	Y	Y	.	.	.	.	.	.	Y	Y	Y	Y	Y	Y	.	.
	683	N	.	.	.	.	.	.	.	.	.	.	.	.	K	K	K	K	K	K	.	.
	685	P	H	H	.	.	.	.	R	R	.	.	H	H	H	H	H	H	H	H	H	H
	705	A	.	.	V	V	.	.	.	.	.	.	.	.	.	.	.	.	.	.	.	.
	708	S	.	.	.	.	.	.	.	.	.	.	.	.	.	.	.	.	.	.	.	L
	720	T	I	I	.	.	.	.	.	.	.	.	.	.	.	.	.	.	.	.	.	.
	768	N	.	.	.	.	.	.	.	.	.	.	.	.	K	K	K	K	K	K	.	.
	800	D	.	.	.	.	.	.	.	.	.	.	.	.	Y	Y	Y	Y	Y	Y	.	.
	860	N	.	.	.	.	.	.	.	.	.	.	.	.	.	K	.	.	.	K	.	.
	863	T	.	.	.	.	.	.	.	.	N	N	.	.	.	.	.	.	.	.	N	N
	940	D	.	.	.	.	.	.	.	.	.	.	.	.	.	.	.	.	.	.	H	H
	954	D	.	.	.	.	.	.	.	N	.	.	N	N	.	.	.	.	.	.	.	.
	958	Q	.	.	.	.	.	.	.	.	.	.	.	.	H	H	H	H	H	H	.	.
	973	N	.	.	.	.	.	.	.	.	.	.	.	.	K	K	K	K	K	K	.	.
	985	L	.	.	.	.	.	.	.	.	.	.	.	.	.	F	.	.	.	F	.	.
	986	S	A	A	.	.	.	.	.	.	.	.	.	.	.	.	.	.	.	.	.	.
	1031	T	.	.	.	.	I	I	.	.	.	.	.	.	.	.	.	.	.	.	.	.
	1122	D	H	H	.	.	.	.	.	.	.	.	.	.	.	.	.	.	.	.	.	.
	1180	V	.	.	.	.	F	F	.	.	.	.	.	.	.	.	.	.	.	.	.	.
	1240	C	.	.	.	.	.	.	.	.	.	.	.	.	F	.	.	.	.	.	.	.
NP	3	D	L	L	.	.	.	.	.	.	.	.	.	.	.	.	.	.	.	.	.	.
	13	P	.	.	.	.	.	.	.	.	L	L	.	.	L	L	L	L	.	.	.	.
	22	D	.	.	.	.	.	.	.	.	.	.	.	.	.	.	.	.	.	.	Y	Y
	31	E	.	.	.	.	.	.	.	.	.	.	.	.	-	-	-	-	.	.	.	.
	32	R	.	.	.	.	.	.	.	.	.	.	.	.	-	-	-	-	.	.	.	.
	33	S	.	.	.	.	.	.	.	.	.	.	.	.	-	-	-	-	.	.	.	.
	63	D	.	.	.	.	.	.	.	G	.	.	.	.	.	.	.	.	G	G	.	.
	80	P	.	.	.	.	R	R	.	.	.	.	.	.	.	.	.	.	.	.	.	.
	203	R	K	K	.	.	K	K	M	M	K	K	.	.	K	K	K	K	K	M	.	.
	204	G	R	R	.	.	R	R	.	.	R	R	.	.	R	R	R	R	R	.	.	.
	205	T	.	.	I	I	.	.	.	.	.	.	I	I	.	.	.	.	.	.	I	I
	214	G	.	.	.	.	.	.	.	.	C	C	.	.	.	.	.	.	.	.	.	.

	215	G	.	.	.	.	.	.	.	.	.	.	.	.	.	.	.	.	.	C	.	.
	235	S	F	F	.	.	.	.	.	.	.	.	.	.	.	.	.	.	.	.	.	.
	273	A	S	.	.	.	.	.	.	.	.	.	.	.	.	.	.	.	.	.	.	.
	300	H	.	.	.	.	.	.	.	.	.	.	.	.	.	.	L	.	.	.	.	.
	366	T	.	.	.	.	.	.	.	.	I	I	.	.	.	.	.	.	.	.	.	.
	377	D	.	.	.	.	.	.	Y	Y	.	.	.	.	.	.	.	.	.	Y	.	.
	378	E	.	.	.	.	.	.	.	.	.	.	.	.	.	.	.	.	.	.	Q	Q
	385	R	.	.	.	.	.	.	K	.	.	.	.	.	.	.	.	.	.	.	.	.
	413	S	.	.	.	.	.	.	.	.	.	.	.	.	R	.	R	R	R	.	.	.
MA	3	D	.	.	.	.	.	.	.	.	.	.	.	.	G	.	.	.	.	.	.	
	19	Q	.	.	.	.	.	.	.	.	.	.	.	E	E	E	E	E	.	.	.	
	63	A	.	.	.	.	.	.	.	.	.	.	.	T	T	T	T	T	.	.	.	
	82	I	.	.	.	.	.	T	T	.	.	.	.	.	.	.	.	.	T	T	T	
	183	A	.	.	.	.	.	.	G	.	.	.	.	.	.	.	.	.	.	.	.	
	184	S	.	.	.	.	.	.	-	.	.	.	.	.	.	.	.	.	.	.	.	.
	185	Q	.	.	.	.	.	.	-	.	.	.	.	.	.	.	.	.	.	.	.	.
	186	R	.	.	.	.	.	.	-	.	.	.	.	.	.	.	.	.	.	.	.	.
Protein E	9	T	.	.	.	.	.	.	.	.	.	.	.	I	I	I	I	I	.	.	.	
	71	P	.	L	.	.	.	.	.	.	.	.	.	.	.	.	.	.	.	.	.	
ORF3A	26	S	.	.	.	.	.	.	L	L	.	.	.	.	.	.	.	.	.	.	.	.
	32	T	.	.	.	.	.	.	.	.	.	.	.	.	.	.	.	.	.	.	I	I
	57	Q	.	.	H	H	.	.	.	.	.	.	H	H	.	.	.	.	.	.	H	H
	74	S	.	A	.	.	.	.	.	.	.	.	.	.	.	.	.	.	.	.	.	.
	92	S	.	.	.	.	.	.	.	.	.	.	.	.	.	.	.	.	.	L	.	.
	155	D	.	.	.	.	.	.	.	.	.	.	.	.	.	.	.	.	.	Y	.	.
	166	S	.	.	.	.	.	L	.	.	.	.	.	.	.	.	.	.	.	.	.	.
	171	S	.	.	L	L	.	.	.	.	.	.	.	.	.	.	.	.	.	.	.	.
	223	T	.	.	.	.	.	.	.	.	.	.	.	.	I	.	I	I	I	.	.	.
	253	S	.	.	.	.	P	P	.	.	.	.	.	.	.	.	.	.	.	.	.	.
	256	V	.	.	.	.	.	.	.	.	.	.	I	I	.	.	.	.	.	.	.	.
	257	N	.	.	.	.	.	.	.	.	.	.	Q	Q	.	.	.	.	.	.	.	.
	298	P	.	.	.	.	.	.	.	.	.	.	Stp*	Stp*	.	.	.	.	.	.	.	.
ORF6	61	D	.	.	.	.	.	.	.	.	.	.	.	.	L	.	L	L	L	.	.	.
ORF7A	73	H	.	.	.	.	.	.	.	.	.	.	.	Y	.	.	.	.	.	.	.	.
	82	V	.	.	.	.	.	.	A	A	.	.	.	.	.	.	.	.	.	A	.	.
	116	L	.	.	.	.	.	.	F	.	.	.	.	.	.	.	.	.	.	.	.	.
	120	T	.	.	.	.	.	.	I	I	.	.	.	.	.	.	.	.	.	I	.	.

ORF7B	40	T	.	.	.	.	.	.	.	.	.	.	.	.	.	.	.	.	.	I	.	.
ORF8	8	G	.	.	.	.	.	.	V	.	.	.	.	.	.	.	.	.	.	.	.	.
	11	T	.	.	.	.	.	.	.	.	.	.	K	K	.	.	.	.	.	.	.	.
	38	P	.	.	.	.	.	.	.	.	.	.	S	S	.	.	.	.	.	.	.	.
	52	R	I	I	.	.	.	.	.	.	.	.	.	.	.	.	.	.	.	.	.	.
	67	S	.	.	.	.	.	.	.	.	.	.	F	F	.	.	.	.	.	.	.	.
	73	Y	C	C	.	.	.	.	.	.	.	.	.	.	.	.	.	.	.	.	.	.
	92	E	.	.	.	.	K	K	.	.	.	.	.	.	.	.	.	.	.	.	.	.
	119	D	.	.	.	.	.	.	-	.	.	.	.	.	.	.	.	.	.	-	.	.
	120	F							-	.	.	.	.	.	.	.	.	.	.	-	.	.

Virus strains: WH-1 is Wuhan Hu-1; A-1 is Alpha EPI ISL 1489174; A-2 is Alpha EPI ISL 1489173; B-1 is Beta EPI ISL 660190; B-2 is Beta EPI ISL 660613; G-1 is Gamma EPI ISL 811149; G-2 is Gamma EPI ISL 872192; D-1 is Delta EPI ISL 1544014; D-2 is Delta EPI ISL 1838642; L-1 is Lambda EPI ISL 1111127; L-2 is Lambda EPI ISL 1111341; M-1 is Mu EPI ISL 2339799; M-2 is Mu EPI ISL 2339801; O-1 is Omicron EPI ISL 9010924; O-2 is Omicron EPI ISL 9042199; BA2-1 is BA.2 LSPA-3B08C6A; BA2-2 is BA.2 HSLL-3B08857; GKA-1 is GKA EPI ISL 9021856; GKA-2 is GKA HDF-IPP08027; GH-1 is GH/490 EPI ISL 6316493; GH-2 is GH/490 ISL 7702915;

*Stp: Stop Codon
